# Proof of concept for a superior therapeutic index of corticosterone compared with hydrocortisone in patients with congenital adrenal hyperplasia

**DOI:** 10.1093/ejendo/lvae144

**Published:** 2024-11-15

**Authors:** Catriona J Kyle, Luke D Boyle, Mark Nixon, Natalie Z M Homer, Joanna P Simpson, Alison Rutter, Lynne E Ramage, Alexandra Kelman, Ellen Marie Freel, Ruth Andrew, Brian R Walker, Roland H Stimson

**Affiliations:** University/BHF Centre for Cardiovascular Science, Queen's Medical Research Institute, University of Edinburgh, EH16 4TJ, United Kingdom; University/BHF Centre for Cardiovascular Science, Queen's Medical Research Institute, University of Edinburgh, EH16 4TJ, United Kingdom; University/BHF Centre for Cardiovascular Science, Queen's Medical Research Institute, University of Edinburgh, EH16 4TJ, United Kingdom; University/BHF Centre for Cardiovascular Science, Queen's Medical Research Institute, University of Edinburgh, EH16 4TJ, United Kingdom; University/BHF Centre for Cardiovascular Science, Queen's Medical Research Institute, University of Edinburgh, EH16 4TJ, United Kingdom; University/BHF Centre for Cardiovascular Science, Queen's Medical Research Institute, University of Edinburgh, EH16 4TJ, United Kingdom; University/BHF Centre for Cardiovascular Science, Queen's Medical Research Institute, University of Edinburgh, EH16 4TJ, United Kingdom; University/BHF Centre for Cardiovascular Science, Queen's Medical Research Institute, University of Edinburgh, EH16 4TJ, United Kingdom; Queen Elizabeth University Hospital, Glasgow, G51 4TF, United Kingdom; University/BHF Centre for Cardiovascular Science, Queen's Medical Research Institute, University of Edinburgh, EH16 4TJ, United Kingdom; University/BHF Centre for Cardiovascular Science, Queen's Medical Research Institute, University of Edinburgh, EH16 4TJ, United Kingdom; Translational and Clinical Research Institute, Newcastle University, Newcastle upon Tyne, NE4 5PL, United Kingdom; University/BHF Centre for Cardiovascular Science, Queen's Medical Research Institute, University of Edinburgh, EH16 4TJ, United Kingdom

**Keywords:** congenital adrenal hyperplasia, corticosterone, hydrocortisone, androgens, insulin resistance

## Abstract

**Objective:**

Outcomes are poor for patients with congenital adrenal hyperplasia (CAH), in part due to the supraphysiological glucocorticoid doses required to control adrenal androgen excess. Hydrocortisone (ie, cortisol) is the recommended glucocorticoid for treatment of CAH. However, the other endogenous glucocorticoid in humans, corticosterone, is actively transported out of metabolic tissues such as adipose tissue and muscle, so we hypothesized that corticosterone could control adrenal androgens while causing fewer metabolic adverse effects than hydrocortisone.

**Methods:**

Thirteen patients (8 female, 5 male) with CAH due to 21-hydroxylase deficiency completed a randomized placebo-controlled crossover study comparing 5 h intravenous infusions of either hydrocortisone, corticosterone or placebo. 6-6[^2^H]_2_-glucose and 1,1,2,3,3-[^2^H]_5_-glycerol were infused to measure glucose and glycerol kinetics, and blood samples were collected throughout. Subcutaneous abdominal adipose tissue biopsies were obtained at the end of each infusion.

**Results:**

During the infusion, corticosterone and hydrocortisone similarly reduced ACTH, 17α-hydroxyprogesterone, androstenedione, and testosterone (in females only) compared with placebo. Despite achieving circulating corticosterone concentrations ∼2.5-fold higher than hydrocortisone, by T + 300 min hydrocortisone but not corticosterone increased glucose and insulin concentrations and reduced 6-6-[^2^H]_2_-glucose clearance compared with placebo. Hydrocortisone increased mRNA levels of the glucocorticoid regulated transcript *PER1* in adipose to a greater extent than corticosterone.

**Conclusions:**

Corticosterone acutely controls biochemical markers of androgen excess similarly to hydrocortisone but without inducing markers of glucocorticoid “toxicity” in CAH. These data demonstrate proof of concept that corticosterone may be a safer glucocorticoid replacement than current medications, although further research is required to assess the longer-term effects of corticosterone replacement.

**Significance**

Outcomes for patients with congenital adrenal hyperplasia are poor, in part due to the toxicity of current glucocorticoid replacement strategies. In this study, we demonstrate proof of concept that corticosterone may be a metabolically safer glucocorticoid in patients with classic CAH due to 21-hydroxylase deficiency. Corticosterone infusion over 5 h suppressed biochemical markers of disease control to a similar extent as hydrocortisone, but did not increase glucose and insulin concentrations unlike hydrocortisone. Further research is needed to determine whether corticosterone's superior therapeutic index is maintained during more chronic treatment.

## Introduction

Congenital adrenal hyperplasia (CAH) is a group of autosomal recessive disorders characterized by defective adrenal steroidogenesis resulting in deficiency of the glucocorticoid cortisol and in many cases the mineralocorticoid aldosterone.^1^ Over 90% of cases are due to 21-hydroxylase deficiency secondary to inactivating mutations in *CYP21A2.*^2,3^ The resulting lack of glucocorticoid (and mineralocorticoid) activates the hypothalamic-pituitary-adrenal (HPA) axis causing excessive synthesis of adrenocorticotrophic hormone (ACTH) and adrenal androgens. Current treatment involves replacement of glucocorticoid (and mineralocorticoid) deficiency with the additional aim of reducing the HPA axis activity and androgen excess. Current guidelines for treating 21-hydroxylase deficiency recommend hydrocortisone (ie, a pharmaceutical preparation of cortisol) as the preferred glucocorticoid, although other longer acting synthetic glucocorticoids such as prednisolone and dexamethasone are also commonly used in adults.^4^ However, outcomes for patients with CAH are poor with increased rates of obesity, adiposity, cardiometabolic disease, osteoporosis, and impaired quality of life.^5-9^ Supraphysiological glucocorticoid doses are often required to normalize androgens, and toxicity from glucocorticoid treatment regimens is a contributing factor to these poor outcomes.^10,11^ Chronic glucocorticoid excess also increases cardiovascular risk factors and arterial stiffness.^12^ However, adrenal crisis is a major cause of premature mortality in these patients so adequate steroid replacement is crucial.^13^

In the past 10 years, several novel approaches to treat CAH have been suggested, most commonly by altering the pharmacokinetics of glucocorticoid replacement. The furthest developed of these is delayed/modified release hydrocortisone, which can achieve a more physiological cortisol profile to prevent the ACTH-induced elevation of adrenal androgens in early morning, reducing concentrations of the precursors 17α-hydroxprogesterone and androstenedione.^14,15^ Continuous subcutaneous hydrocortisone infusion (CSHI) can also reduce biochemical markers of androgen excess by achieving a more physiological cortisol profile,^16^ although improved disease control may be at the expense of signs of glucocorticoid excess such weight gain, insulin resistance, and reduced bone density with increased levels of the bone turnover marker osteocalcin.^14,16^ However, optimization of CSHI over time may reduce the total glucocorticoid dose and the adverse effects due to glucocorticoid excess.^17^ An alternative approach is to try to block ACTH-stimulated androgen synthesis, for example using inhibitors of the corticotrophin-releasing factor type 1 receptor (CRFR1),^18^ the melanocortin 2 (ACTH) receptor^19^ or key enzymes in the androgen synthesis pathway such as CYP17A1.^20^ Normalization of androgens using such compounds could then result in lower glucocorticoid replacement doses to minimize the risk of complications due to glucocorticoid excess.

All of these approaches rely on the use of hydrocortisone or other longer acting glucocorticoids currently available. However, another solution is to identify a metabolically safer glucocorticoid, one that can suppress ACTH to normalize androgens but with less toxicity in key metabolic tissues such as adipose tissue and muscle. While cortisol is the primary glucocorticoid, humans also synthesize corticosterone. Although corticosterone accounts for only ∼5% of circulating glucocorticoid levels in humans, it accounts for ∼25% of glucocorticoids in the brain.^21,22^ This is due to active export of glucocorticoids by two members of the ATP-binding cassette (ABC) family of transporters (reviewed by Devine et al.^23^). ABCB1 (also known as multidrug resistance protein 1 (MDR1)) is highly expressed in the brain and blood–brain barrier and exports cortisol, prednisolone, and dexamethasone but not corticosterone from the brain, explaining the high corticosterone concentrations in the CNS.^22^ Conversely, ABCC1 (also called multidrug resistance-associated protein 1 (MRP1)) is expressed in tissues such as adipose tissue and muscle, and exports corticosterone but not cortisol or other synthetic glucocorticoids out of cells.^24,25^ Mice lacking ABCC1 accumulate greater corticosterone in adipose tissue than control littermates.^25^ In humans, corticosterone infusion suppressed ACTH similarly to hydrocortisone in patients with Addison's disease, without mimicking hydrocortisone's effect to increase transcript levels of the glucocorticoid-responsive genes *PER1* and *LPL* in subcutaneous abdominal adipose tissue.^25^ These data suggest that corticosterone may be a metabolically safer glucocorticoid than hydrocortisone, with similar effects on the brain but reduced action in peripheral metabolic tissues, which may confer particular benefit to patients with CAH who are often treated with supraphysiological hydrocortisone doses. We hypothesized that corticosterone would suppress adrenal androgens similarly to hydrocortisone but cause less metabolic disturbance in patients with CAH. Due to lack of an oral preparation of corticosterone, we tested this utilizing intravenous infusion.

## Methods

### Study protocol

Fourteen patients were recruited to this randomized double-blind placebo-controlled crossover study comparing the effects of corticosterone and hydrocortisone. Local ethical approval was obtained (ethics number 16/SS/0045) as was written consent from each participant, in accordance with the Declaration of Helsinki. Patients were recruited from the endocrine clinics in either Edinburgh or Glasgow, where they received their regular clinical care. Inclusion criteria were as follows: aged 18-80 years of age; male or female gender; confirmed clinical diagnosis of classic CAH secondary to 21-hydroxylase deficiency; receiving oral glucocorticoid therapy for CAH; not receiving additional glucocorticoid treatment (including topical or inhaled) for unrelated conditions; no admissions with adrenal crisis in the preceding 6 months; blood pressure >90/50 mm Hg; screening blood tests within acceptable limits (blood count, kidney, liver, and thyroid function and random glucose). Each participant's daily hydrocortisone equivalent dose was calculated using the ratio of hydrocortisone:prednisolone:dexamethasone of 4:1:0.15, respectively, as previously described.^10^ Thereafter, participants attended the Edinburgh Clinical Research Facility (ECRF) at 08:00 h following a 10-h fast on 3 separate occasions, each separated by at least 3 weeks. Participants were randomized to receive intravenous infusions of either placebo, hydrocortisone (cortisol) or corticosterone at each visit. Due to lack of an acceptable preparation of endogenous corticosterone, 2,2,4,6,6,17,21,21[^2^H]_8_-corticosterone (D8-corticosterone) (Cambridge Isotope Laboratories, Massachusetts) was used following filter sterilization. Due to a randomization error one of the participants received the same infusion on two occasions so their data were removed from the analyses as they did not complete all 3 phases.

**Figure 1.**
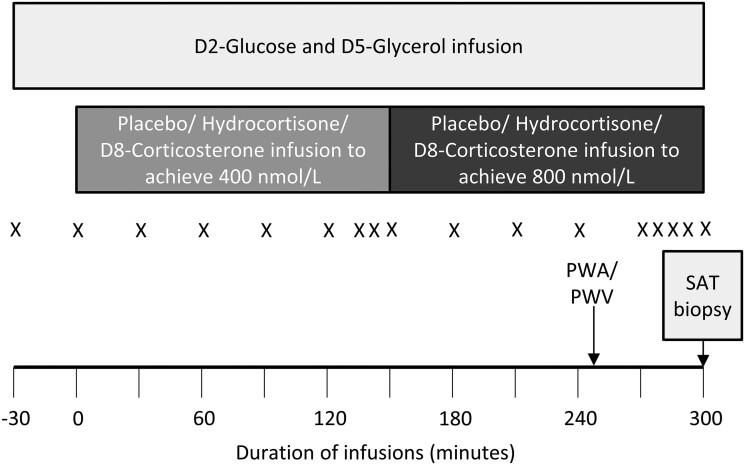
Study visit protocol. Participants were randomized to receive an intravenous infusion of either hydrocortisone, D8-corticosterone, or placebo at each visit in a crossover design. At T-30 min, an intravenous infusion of D2-glucose and D5-glycerol was commenced. At T = 0 min, the glucocorticoid/placebo infusion was commenced for 5 h, aiming to achieve circulating glucocorticoid concentrations of 400 nmol/L in the first 150 min and 800 nmol/L in the final 150 min. Blood pressure was performed at T = 0 and at T + 300 min. Blood samples were obtained regularly (X) throughout the study visit. PWA and PWV were measured at T + 250 min. At T + 300 min, an abdominal subcutaneous adipose tissue (SAT) biopsy was obtained.

The day prior to each visit, participants were advised to administer their prescribed glucocorticoid and mineralocorticoid replacement doses but to omit any glucocorticoid dose from 18:00 h onwards including the following morning. On the day of each visit, volunteers attended the ECRF at 08:00 h following a 10-h fast. Measurements were performed of height, weight, blood pressure (by sphygmomanometer), and fat mass by bioimpedance (using an Omron BF-302). Two cannulae were inserted, one in the antecubital fossa for infusions and another retrogradely in a dorsal vein of the contralateral hand for blood sampling. The hand was placed in a box heated to 55-60 °C for 5 min prior to each time point to obtain arterialized blood samples. At T-30 min (see Figure 1), intravenous infusions of 6,6-[^2^H]_2_-glucose (D2-glucose, at 0.22 μmol/kg/min following a 17.6 μmol/kg bolus) and 1,1,2,3,3-[^2^H]_5_-glycerol (D5-glycerol, at 0.11 μmol/kg/min following a bolus of 1.66μmol/kg) were commenced for 5 h.^26^ At T = 0 min, an intravenous infusion of either 0.9% sodium chloride (placebo), hydrocortisone (at a rate of 51.5 nmol/min following a 2.6 μmol bolus), or D8-corticosterone (at 336.7 nmol/min following a 7.2 μmol bolus) was commenced for 150 min, designed to achieve a glucocorticoid concentration of ~400 nmol/L.^25^ At T + 150 min, aiming to achieve glucocorticoid concentrations of ∼800 nmol/L, the infusion rate of hydrocortisone was increased to 103 nmol/min (following a 2.6 μmol bolus) while the D8-corticosterone infusion rate was increased to 673.4 nmol/min (following a 7.2 μmol bolus). On the placebo phase, a further bolus of 0.9% saline was given and the infusion rate doubled accordingly.

Blood sampling was performed as detailed in Figure 1. Blood pressure was measured at T = 0 and at T + 300 min, while pulse wave analysis (PWA) and pulse wave velocity (PWV) were measured at T + 250 min. A subcutaneous abdominal fat biopsy was obtained at T + 300 min as described previously.^27^ Following completion of the biopsy, the infusions were discontinued, participants were given their glucocorticoid medication and allowed home.

### Biochemical analyses

Plasma glucocorticoids (cortisol and D8-corticosterone), androgens and precursors (testosterone, androstenedione, and 17α-hydroxyprogesterone (17OHP)) were quantified by liquid chromatography tandem mass spectrometry (LC-MS/MS) using a panel version of our targeted steroid profiling chromatography and mass spectrometry method^28^ to include D8-corticosterone as an analyte. Further information including validation of this assay is detailed in the supplemental methods and in Tables S1 and S2. Endogenous and tracer glucose and glycerol were analyzed as previously described following method transfer to a gas chromatography tandem mass spectrometer.^29^ ELISAs were used to measure plasma ACTH (Biomerica, Irvine, CA), serum insulin (DRG Diagnostics, Marburg, Germany), and osteocalcin (N-MID®, IDS, Boldon, UK). Serum nonesterified fatty acids (NEFAs) were quantified by colorimetric assay (Wako Chemicals, Germany).

### Adipose tissue quantitative real time PCR measurements

RNA extraction and qPCR was performed as described previously.^30^ The gene-specific primers (Invitrogen) and fluorescent probes (Roche Universal Probe Library) are detailed in Table S3. Transcript levels are presented as the ratio of the abundance of the gene of interest: mean of abundance of control genes (*PPIA* and *RNA18S5*).

### PWA and PWV

PWA was performed at the radial artery by applanation tonometry using a SphygmoCor device (AtCor Medical Inc, Illinois, USA). The radial pulse waveform was recorded and central aortic pressure derived using an automated generalized transfer function.^31^ The augmentation index was calculated as the increment in pressure from the shoulder of the ascending pressure wave to the peak of the reflected wave. To correct for the effect of pulse rate, augmentation index results were normalized for a heart rate of 75 bpm.

To calculate carotid-femoral PWV, the distance between the carotid and femoral arterial capture sites and the sternal notch were measured. Applanation tonometry was performed at each site and the arterial waveform recorded. Simultaneous electrocardiogram monitoring was performed and the R-wave was used as a reference point for PWV calculations. PWV was calculated from the difference in transit time of the pulse wave to the carotid and femoral arteries.

### Glucose and glycerol kinetic analyses

Analysis of steady state (ss) measurements was performed using the mean of the 5 samples obtained between T + 270 and T + 300 min. The metabolic clearance of glucose was assessed using eqn (1):


(1) $$\begin{array}{rl} & \mathrm{Metabolic}\;\mathrm{clearance}\;\mathrm{of}\;\mathrm{glucose}\;(L/\min)=\\ & D2-\mathrm{Glucose}\;\mathrm{infusion}\;\mathrm{rate}/D2-\mathrm{Glucose}\;\mathrm{concentratio}n_{\mathrm{ss}} \end{array}$$

Steele's steady state equation^32^ was used to measure the rate of appearance (Ra) of endogenous glucose and glycerol respectively as described in eqn (2), where TTR_ss_ is the tracer:tracee ratio (eg, D2-glucose/glucose) at steady state:


(2) $$\begin{array}{rl} \mathrm{Ra}\;\mathrm{Glucose}\;= & D2-\mathrm{Glucose}\;\mathrm{infusion}\;\mathrm{rate}/\\ & D2-\mathrm{Glucose}\;\mathrm{TT}R_{\mathrm{ss}} \end{array}$$

### Statistical analyses

Data are presented as mean ± SEM unless otherwise stated. All data were analyzed using SPSS version 27. Comparisons between the phases were analyzed by repeated measures ANOVA, with post hoc LSD testing. Paired comparisons over time were tested either by 2-way repeated measures ANOVA or by linear mixed effects models with post hoc LSD testing. *P* < .05 was considered significant.

## Results

Participant characteristics are shown in Table 1; mean age was 34.7 ± 3.4 years with a BMI of 31.5 ± 2.9 kg/m^2^. The median daily hydrocortisone equivalent dose of the 13 volunteers was 20 mg.

**Table 1.** Subject characteristics.

	Mean	Range
Participants	13 (8 Female, 5 Male)	
Age (years)	34.7 ± 12.4	19-55
Weight (kg)	80.5 ± 25.2	47.1-133.3
Height (m)	1.60 ± 0.08	1.44-1.69
Body mass index (kg/m^2^)	31.5 ± 10.6	20.7-52.7
Body fat (%)	31.2 ± 7.5	19.3-42.4
**Glucocorticoid medication**		
No. taking hydrocortisone	7	
No. taking prednisolone	7	
No. taking dexamethasone	1	
Median hydrocortisone equivalent dose (mg/day)	20	15-30
**Mineralocorticoid medication**		
No. taking fludrocortisone	10	
Median fludrocortisone dose (mcg/day)	162.5	100-400

Data are mean ± SD unless otherwise indicated for the 13 participants who completed the study. Two participants were prescribed hydrocortisone during the day and prednisolone before bed. Hydrocortisone equivalent dose was calculated using the following equation: 20 mg hydrocortisone = 5 mg prednisolone = 0.75 mg dexamethasone. The median fludrocortisone dose excludes the patients not prescribed regular fludrocortisone. Bioimpedance was not possible on one patient so the data presented are from *n* = 12.

### Glucocorticoid concentrations during infusions

**Figure 2.**
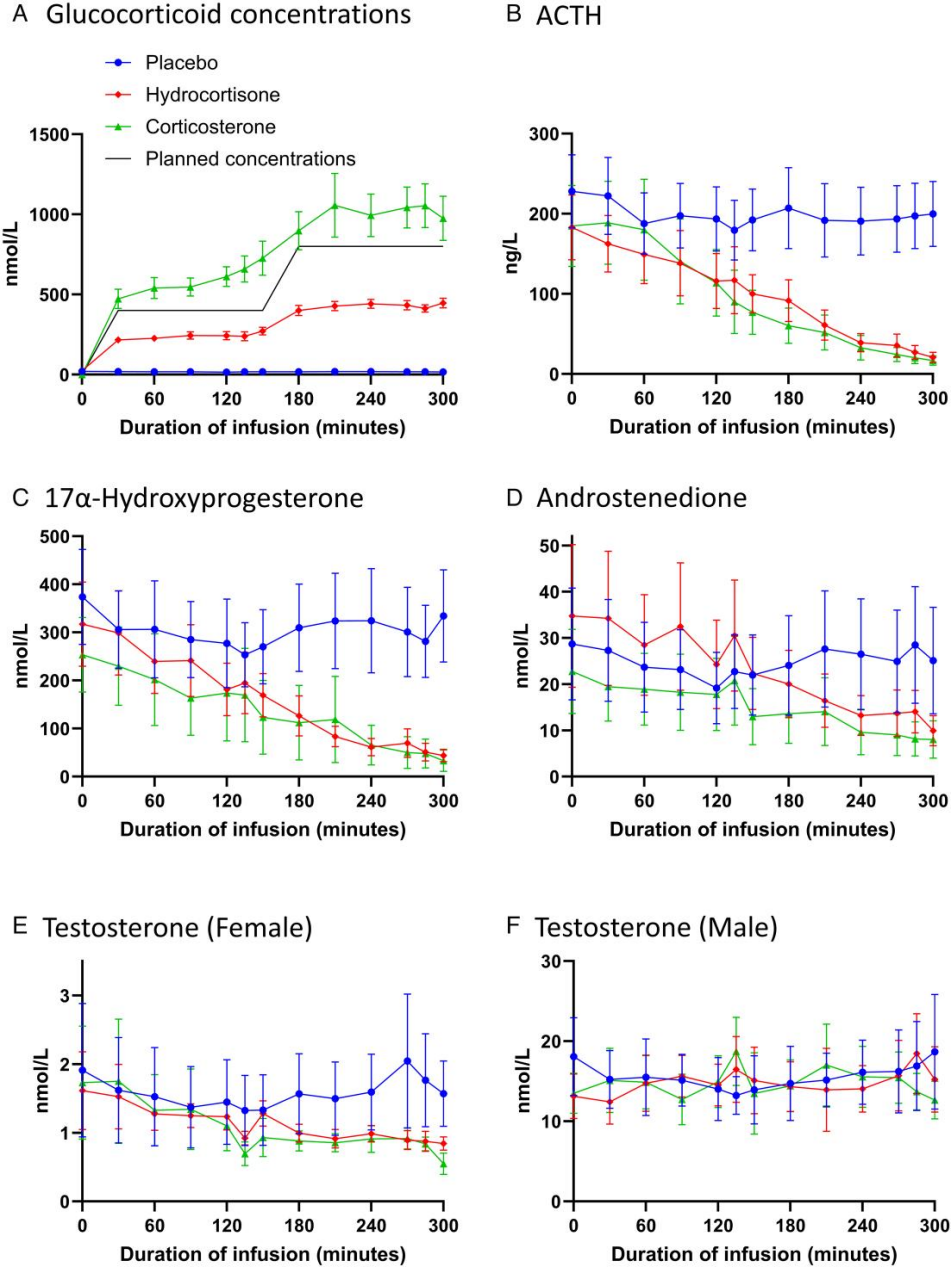
Effect of hydrocortisone and corticosterone on measures of disease control. Data are mean ± SEM for (A) circulating glucocorticoid (*n* = 13), (B) ACTH, (C) 17OHP, (D) androstenedione (all *n* = 10), and (E/F) testosterone concentrations in (E) female (*n* = 6), and (F) male (*n* = 5) participants. (A) Cortisol concentrations on placebo (blue circles/lines) and hydrocortisone (red diamonds/lines) phases, along with D8-corticosterone concentrations (green triangles/lines) on the corticosterone phase, with planned circulating glucocorticoid concentrations shown (black line). (B-E) Hydrocortisone and corticosterone decreased all measurements of disease control compared with placebo, but to a similar degree between both glucocorticoid phases. (F) Testosterone concentrations were similar on all 3 phases in males. Data were analyzed by linear mixed model with post hoc LSD testing.

Mean fasting cortisol concentrations were 20 ± 5 nmol/L on the placebo phase, and levels remained low throughout the infusion (see Figure 2A). While planned circulating glucocorticoid concentrations on the 2 active phases were ∼400 nmol/L (up to T + 150 min) and ∼800 nmol/L (up to T + 300 min), hydrocortisone concentrations were lower than D8-corticosterone (Figure 2A, *P* < .001). During the hydrocortisone phase mean circulating cortisol concentrations were lower than anticipated (∼250 and ∼430 nmol/L, respectively). Conversely, during the D8-corticosterone infusion, circulating D8-corticosterone concentrations were higher than planned (∼650 and ~1000 nmol/L, respectively).

### Effect of hydrocortisone and corticosterone on circulating ACTH and androgens

In 3 of the volunteers (2 female and 1 male), ACTH, 17OHP, androstenedione and testosterone (in female patients) were below the limit of detection at baseline and during the infusions during all 3 phases, in keeping with suppression of the HPA axis. Therefore, the effect of hydrocortisone and D8-corticosterone (hereafter termed corticosterone) on biochemical measures of control was assessed only in the 10 participants with detectable ACTH, 17OHP and androstenedione concentrations. Despite the substantial difference in circulating glucocorticoid concentrations, hydrocortisone and corticosterone reduced ACTH (Figure 2B) and 17OHP (Figure 2C) (both *P* < .001 vs placebo), androstenedione (Figure 2D) (*P* < .01 for corticosterone and *P* < .05 for hydrocortisone vs placebo), and testosterone (Figure 2E) (*P* < .05 vs placebo only in female participants). None of these measures differed between hydrocortisone and corticosterone phases. Compared with baseline, by T + 300 min hydrocortisone and corticosterone reduced ACTH and 17OHP concentrations by ∼80%–90% (Figure 2B and C) and androstenedione and testosterone (in female participants only) by ∼50%–60% (Figure 2D and E). In male patients, neither corticosterone nor hydrocortisone significantly reduced testosterone concentrations compared with placebo (Figure 2F). ACTH, adrenal intermediates, and androgen concentrations did not change throughout the placebo infusion (Figure 2B–F).

### Effect of hydrocortisone and corticosterone on cardiometabolic measurements

**Figure 3.**
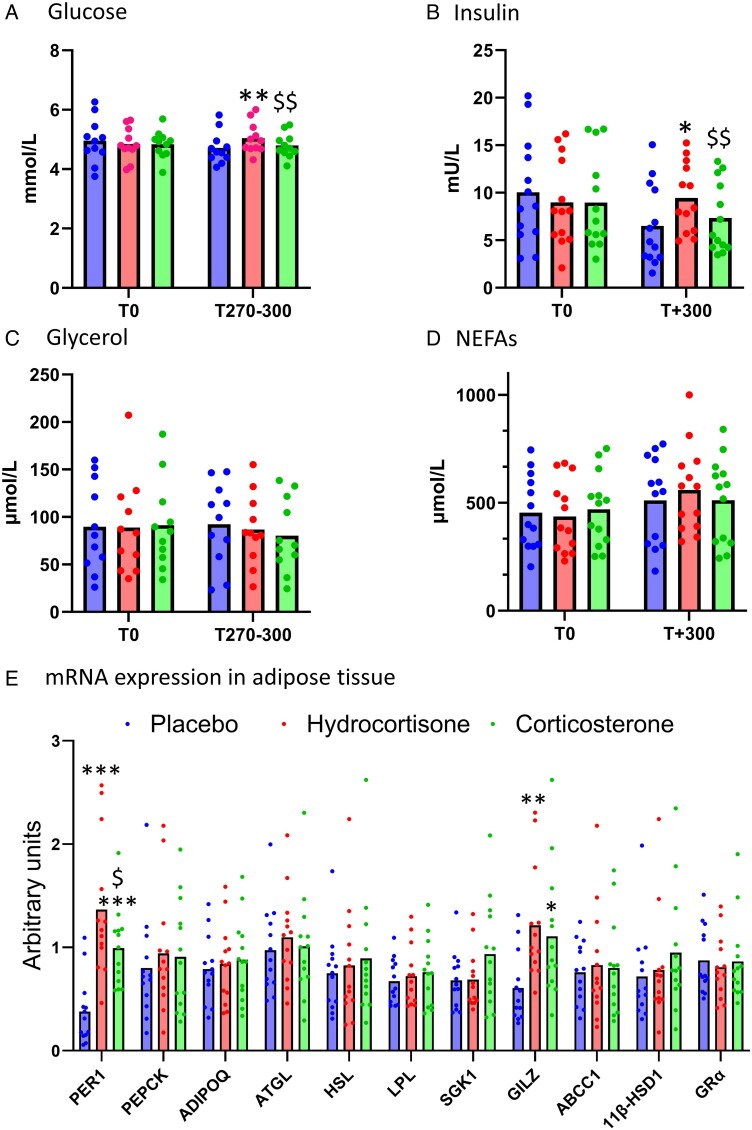
Hydrocortisone but not corticosterone increases glucose and insulin concentrations. Data are mean ± SEM for circulating (A) glucose, (B) insulin, (C) glycerol, and (D) NEFAs prior to and following placebo (blue dots/columns), hydrocortisone (red), and corticosterone (green) infusions. Data for glucose and glycerol are the mean values taken during steady state (T + 270-300) while for insulin and NEFAs values were obtained at T + 300 min. Hydrocortisone increased circulating glucose and insulin concentrations. (E) qPCR data from abdominal adipose tissue, both hydrocortisone and corticosterone increased expression of PER1 and GILZ vs placebo. Data were analyzed by repeated measures ANOVA with post hoc LSD testing. *P < .05, **P < .01, ***P < .001 vs placebo; $*P* < .05, $$*P* < .01 vs hydrocortisone. 11β-HSD1, 11β-hydroxysteroid dehydrogenase type 1; ADIPOQ, adiponectin; ATGL, adipose triglyceride lipase; GILZ, glucocorticoid-induced leucine zipper; GRα, glucocorticoid receptor-α; HSL, hormone sensitive lipase; LPL, lipoprotein lipase; PEPCK, phosphoenolpyruvate carboxykinase; PER1, period circadian regulator 1; SGK1, serum/glucocorticoid regulated kinase 1.

Prior to the infusions, plasma glucose and glycerol, serum insulin, and NEFA concentrations were similar between the 3 phases (Figure 3). By the end of the infusions, plasma glucose was higher on hydrocortisone compared with both placebo and corticosterone phases (Figure 3A). Similarly, hydrocortisone reduced the metabolic clearance rate of D2-glucose at steady state compared with placebo, but did not alter the rate of appearance of glucose (Table 2). Hydrocortisone also increased serum insulin compared with placebo and corticosterone phases (Figure 3B). Despite achieving D8-corticosterone concentrations ∼2.5-fold higher than hydrocortisone, corticosterone did not increase glucose or insulin concentrations compared with placebo. Neither hydrocortisone nor corticosterone increased glycerol or NEFA concentrations (Figure 3C and D) or the rate of appearance of glycerol at steady state (Table 2). mRNA transcript levels of known glucocorticoid-regulated genes were measured in subcutaneous abdominal adipose tissue at the end of each phase. Both hydrocortisone and corticosterone infusions increased expression of *PER1* and *GILZ* vs placebo, but hydrocortisone increased *PER1* mRNA levels to a greater extent than corticosterone (Figure 3E, *P* < .05). Neither glucocorticoid altered expression of *PCK1*, *ADIPOQ*, *PNPLA2*, *LIPE*, *LPL*, *SGK1*, *ABCC1*, *HSD11B1*, or *NR3C1* in adipose.

**Table 2.** Effect of hydrocortisone and corticosterone on other measurements.

Measurement	Placebo	Hydrocortisone	Corticosterone
Systolic blood pressure (mm Hg)	121 ± 4	119 ± 3	119 ± 3
Diastolic blood pressure (mm Hg)	75 ± 3	71 ± 4	72 ± 4
PWA (augmentation index)	18.5 ± 3.1	17.1 ± 2.2	16.8 ± 1.8
PWV (m/s)	5.8 ± 0.3	5.6 ± 0.3	5.8 ± 0.4
Osteocalcin (ng/mL)	26.0 ± 2.9	27.4 ± 3.0	24.9 ± 2.4
Steady state kinetic measurements			
D2-Glucose metabolic clearance rate (L/min)	0.17 ± 0.02	0.15 ± 0.02^a^	0.16 ± 0.03
Ra glucose (µmol/kg/min)	11.6 ± 2.2	11.4 ± 2.1	11.3 ± 2.2
Ra glycerol (µmol/kg/min)	5.9 ± 0.9	6.3 ± 1.4	5.6 ± 1.1

Data are mean ± SEM for data obtained near or at the end of the infusion on each phase, including blood pressure and serum osteocalcin (obtained at T + 300 min, *n* = 13/phase), PWA/PWV (obtained at T + 250 min, *n* = 10 due to technical failure in 3 participants), and glucose and glycerol kinetics (obtained between T + 270-300 of infusion, *n* = 11 due to technical failure in 2 participants). Augmentation index was normalized to a heart rate of 75 bpm. Data were analyzed by repeated measures ANOVA with post hoc LSD testing.

^a^ *P* < .05 vs placebo.

Neither glucocorticoid altered blood pressure, PWA/PWV, or circulating osteocalcin concentrations compared with placebo (Table 2).

## Discussion

These data demonstrate that the endogenous glucocorticoid corticosterone can normalize biochemical markers of serum androgens in patients with 21-hydroxylase deficiency. Furthermore, the circulating concentrations of D8-corticosterone and hydrocortisone were equipotent for suppression of ACTH, 17OHP, androstenedione, and testosterone concentrations, but had contrasting effects on circulating glucose and insulin concentrations and adipose tissue gene expression. This demonstrates proof of concept that corticosterone may induce less metabolic toxicity while maintaining efficacy in the treatment of CAH. These data are consistent with our hypothesis based on the tissue distribution of ABCC1 (that exports corticosterone) and ABCB1 (that exports cortisol, prednisolone and dexamethasone), with lower ABCC1 expression in the majority of the regions of the brain including hypothalamus and hippocampus but higher expression in adipose tissue, skeletal muscle, and cardiac muscle.^23,33^ Therefore, ABCC1 will protect adipose tissue and muscle by reducing intracellular corticosterone concentrations, while having a lesser effect on brain corticosterone levels leading to a proportionally greater effect of corticosterone in central than peripheral tissues.

We aimed to achieve circulating glucocorticoid concentrations of ∼400 and ∼800 nmol/L during the 2-step infusion protocol and based the infusion rates on our previous study in patients with Addison's disease where we achieved circulating cortisol and D8-corticosterone concentrations of ∼400 nmol/L.^25^ Despite using double the hydrocortisone infusion rate of the previous study, mean circulating cortisol concentrations were well below target at ∼400 nmol/L, while D8-corticosterone concentrations were above target at ∼1000 nmol/L. The reasons for this discrepancy are unclear; while increased cortisol clearance may explain this observation D8-corticosterone levels were not lower than anticipated and the half-life of hydrocortisone in patients with CAH is not reduced.^34^ Nevertheless, despite ∼2.5-fold higher D8-corticosterone than cortisol concentrations, both compounds induced almost identical reductions in ACTH, 17OHP, androstenedione and testosterone concentrations. This suggests either that corticosterone is less potent at suppressing ACTH than cortisol (despite having similar affinities for the glucocorticoid and mineralocorticoid receptors and corticosteroid-binding globulin^35-37^), or that there is a threshold effect and the hydrocortisone concentrations achieved (∼250 nmol/L in the first half of the infusion) were sufficient to suppress ACTH secretion. There is some evidence that ABCC1 also regulates the HPA axis in humans, potentially due to high expression in the pituitary gland, which might reduce the sensitivity of ACTH secretion to corticosterone.^33^ However, in support of the latter explanation, previous data from subcutaneous hydrocortisone infusions demonstrate that similar cortisol concentrations successfully suppress ACTH in patients with 21-hydroxylase deficiency.^16,38^

The similar suppression of androgens allows for a direct comparison of the toxicities between the two glucocorticoids. Despite a short infusion protocol of 5 h, physiological hydrocortisone concentrations of ∼400 nmol/L were sufficient to increase insulin and glucose concentrations and decrease glucose clearance, in keeping with induction of insulin resistance over this time frame. Despite ∼2.5-fold higher concentrations, corticosterone did not increase these metabolic indices. We also measured transcripts of known glucocorticoid-regulated genes in adipose tissue to assess activation of the glucocorticoid receptor (GR). Hydrocortisone increased expression of *PER1* more so than corticosterone while both similarly increased *GILZ* compared to placebo. There was no change in other transcripts measured, but we have previously found *PER1* to be the most acutely responsive gene to changes in glucocorticoid levels/action in human subcutaneous adipose tissue (SAT).^25,33^ These results support enhanced GR activation in adipose during hydrocortisone infusion. However, neither glucocorticoid increased NEFAs nor the rate of appearance of glycerol (measures of lipolysis) compared with placebo, which may also be due to the short infusion duration. The discrepancy in glucose clearance is also consistent with differential effects of hydrocortisone and corticosterone in skeletal muscle. Neither hydrocortisone nor corticosterone altered the rate of appearance of glucose though, ABCB1 expression is higher than ABCC1 in the liver and it is unclear whether cortisol or corticosterone may be more potent in this tissue.^23^

We also address glucocorticoid toxicity in other tissues where the effect of ABCC1 is unknown. ABCC1 expression is higher than ABCB1 in bone marrow and osteoblasts, potentially conferring protection against steroid-induced osteoporosis.^23,39^ In the current study, neither hydrocortisone nor corticosterone altered any of the other measurements of toxicity such as BP, arterial stiffness, lipolysis or the bone marker osteocalcin, but the treatment duration may have been insufficient to induce any change in these measurements. Longer studies are required to determine whether corticosterone treatment has beneficial cardiometabolic outcomes compared to hydrocortisone. Mechanisms other than ABCC1 may also contribute to the beneficial effects of corticosterone compared with hydrocortisone. Polymorphisms in the GR *NR3C1* alter glucocorticoid sensitivity,^40^ including in patients with CAH,^41^ which could theoretically induce differential GR sensitivity to distinct glucocorticoids.

While glucocorticoid over-replacement causes adverse effects, adrenal crisis is an important cause of premature mortality in adults with CAH.^13^ It is unclear whether the risk of adrenal crisis would differ between hydrocortisone and corticosterone, although patients with mutations in *CYP17A1* causing CAH (who sustain high levels of endogenous corticosterone) do not tend to present with adrenal crisis, indicating that corticosterone can protect against adrenal insufficiency at least at the concentrations observed in those patients.^42^ While corticosterone could be used as a novel replacement glucocorticoid, corticosterone has a considerably shorter half-life than cortisol (30-60 min vs 90-120 min),^21,43,44^ meaning multiple doses per day would be required, which would limit compliance. Therefore, it is likely that a modified release preparation would be required similar to those already developed for hydrocortisone.^14,45^ If proven to be a metabolically safer glucocorticoid than current options, corticosterone could be used either as monotherapy or in combination with other novel approaches to reduce androgens such as antagonists to CRFR1 or the ACTH receptor.^1^ In addition, corticosterone could be used to treat other causes of primary and secondary adrenal insufficiency.

Although we have focused on the adverse effects of glucocorticoid treatment in the current paper, it is important to note that it is still unclear to what extent glucocorticoid dosing contributes to the adverse outcomes observed in CAH.^7^ The prevalence of obesity in CAH is increased in some but not all populations,^5,46^ while glucocorticoid (and mineralocorticoid) doses are not always associated with cardiometabolic disease.^9,10,46-48^ Further prospective studies are required to determine the contribution of glucocorticoids to these adverse outcomes, which will be important to determine the cardiometabolic benefits that may be possible from dose reduction.

There are some limitations to the study. As discussed above, the concentrations of corticosterone and cortisol were substantially different, so it was not possible to directly compare the potency of corticosterone and hydrocortisone on ACTH and androgens. In addition, several of the patients included already had suppressed androgens at baseline on each phase, making it impossible to compare the effect of the two glucocorticoids on the measures of disease control that are commonly used in clinical practice. Due to lack of an oral preparation, we infused corticosterone intravenously and it is possible that oral corticosterone may not achieve similar efficacy, for example if bioavailability was reduced compared with hydrocortisone. Importantly, we also infused a labeled form of corticosterone (D8-corticosterone) and it is possible that this isotope may have some differences in biochemical action from endogenous unlabeled corticosterone. Finally, the improved measures of insulin sensitivity in this very short infusion may not be maintained over the long term.

To conclude, we have demonstrated that acute corticosterone infusion can achieve similar HPA axis suppression to hydrocortisone while inducing fewer metabolic abnormalities in patients with CAH due to 21-hydroxylase deficiency. These data provide proof of concept that corticosterone may be a metabolically safer glucocorticoid for replacement in adrenal insufficiency. Further research is required to develop oral preparations of corticosterone in order to test whether corticosterone replacement is a viable therapeutic strategy for CAH and other forms of adrenal insufficiency.

## Supplementary Material

lvae144_Supplementary_Data

## Data Availability

Data are available from the corresponding author upon request.
