# Supplementary material for: Proof of concept for a superior therapeutic index of corticosterone compared with hydrocortisone in patients with congenital adrenal hyperplasia
Source: Eur J Endocrinol. 2024 Nov 15;191(6):535–44. doi: 10.1093/ejendo/lvae144 (PMC11606648; doi:10.1093/ejendo/lvae144)
Supplement: lvae144_Supplementary_Data [file lvae144_supplementary_data.zip › eje-24-0250-File010.docx]

***Table S3 Primer sequences for qPCR and corresponding probe numbers***

*Assays were either performed using the following primer sequences and the relevant Roche probe library or SYBR green as indicated.*

| Gene Name | Primer sequences 5’ to 3’ | Roche UPL Probe number/ SYBR green |
| --- | --- | --- |
| Human samples |  |  |
| *PPIA*  (cyclophilin A) | F: atgctggacccaacacaat | 48 |
|  | R: tctttcactttgccaaacacc |  |
| *RNA18S5*  (18S) | F: cttccacaggaggcctacac | 46 |
|  | R: cgcaaaatatgctggaacttt |  |
| *PER1* | F: ctcttccacagctccctca | 87 |
|  | R: ctttggatcggcagtggt |  |
| *PCK1*  (PEPCK) | F: cgaaagctccccaagtacaa | 20 |
|  | R: gctctctactcgtgccacatc |  |
| *PNPLA2*  (ATGL) | F: ctccaccaacatccacgag | 89 |
|  | R: ccctgcttgcacatctctc |  |
| *LIPE*  (HSL) | F: ggaagtgctatcgtctctgg | SYBR green |
|  | R: ggcagtcagtggcatctc |  |
| *ADIPOQ*  (Adiponectin) | F: ggtgagaagggtgagaaagga | 85 |
|  | R: tttcaccgatgtctcccttag |  |
| *LPL* | F: atgtggcccggtttatca | 25 |
|  | R: ctgtatcccaagagatggacatt |  |
| *SGK1* | F: gcatgcaaacaccctgaag | 25 |
|  | R: aggttgatttgctgagaaggac |  |
| *GILZ* | F: tggtggccatagacaacaag | 10 |
|  | R: tctcggatctgctccttca |  |
| *ABCC1* | F: gcctattaccccagcatcg | 28 |
|  | R: gatgcagttgcccacaca |  |
| *HSD11B1*  (11β-HSD1) | F: caatggaagcattgttgtcg | 20 |
|  | R: ggcagcaaccattggataag |  |
| *NR3C1*  (GRα) | F: ttttcttcaaaagagcagtgga | 11 |
|  | R: gcatgctgggcagttttt |  |
