# Supplementary material for: Proof of concept for a superior therapeutic index of corticosterone compared with hydrocortisone in patients with congenital adrenal hyperplasia
Source: Eur J Endocrinol. 2024 Nov 15;191(6):535–44. doi: 10.1093/ejendo/lvae144 (PMC11606648; doi:10.1093/ejendo/lvae144)
Supplement: lvae144_Supplementary_Data [file lvae144_supplementary_data.zip › eje-24-0250-File007.docx]

**Supplementary Methods – Quantification of cortisol, D8-corticosterone, testosterone, androstenedione and 17α-hydroxyprogesterone by LC-MS/MS**

**Materials and Methods**

All reagents used were of analytical grade or better. Acetonitrile, methanol, water and isopropanol were HPLC grade or better, from VWR. Dichloromethane and formic acid (LC-MS grade) were from Fisher Scientific. Ammonium fluoride was from Sigma-Aldrich. Steroid certified solutions were from Sigma-Aldrich/Cerilliant: Cortisol (C-106) 1 mg/mL in methanol (certified); Testosterone (T-037) 1 mg/mL in acetonitrile (certified); Androstenedione (A4; A-075) 1 mg/mL in acetonitrile (certified); 17α-hydroxyprogesterone (17OHP; H-085) 1 mg/mL in methanol (certified); DC Mass Spect Gold Serum (MSG4000) was also purchased from Sigma-Aldrich and Cerilliant. Internal standards were certified reference material from Sigma-Aldrich/Cerilliant: 9,11,12,12-[^2^H]_4_-Cortisol (D4-cortisol; C-113) 100 μg/mL in methanol; 2,3,4–[^13^C]_3_-Testosterone (^13^C_3_–T; T-070) 100 μg/mL in acetonitrile; 2,3,4–[^13^C]_3_-Androstenedione (^13^C_3_–A4; A-084) 100 μg/mL in acetonitrile; and 2,2,4,6,6,21,21,21-[^2^H]_8_–17α-hydroxyprogesterone (D8-17OHP; H-096) 100 μg/mL in methanol. Isolute SLE+ 400 96-well extraction plates and deep well 96-well collection plates were from Biotage, Uppsala, Sweden. The liquid chromatography column and guard, Kinetex C18 (150 x 2.1 mm; 2.6 µm) and Kinetex Krudcatcher were from Phenomenex, Macclesfield, UK.

**Analytical Methodology**

Steroids were extracted from serum using supported liquid extraction and followed by LC-MS/MS analysis, adapted the method from Devine *et al* ^1^ to a panel version of 5 steroids. A combined 11-point standard curve was prepared for the 5 steroid analytes aligned to expected concentrations of each steroid; cortisol (1.25 – 250 ng/mL), D8-corticosterone (2.5 – 500 ng/mL), testosterone (0.125 – 25 ng), androstenedione (0.125 – 25 ng) and 17α-hydroxyprogesterone (5-125 ng/mL). Plasma samples (200 μL) were enriched with internal standard solution (D4-cortisol, ^13^C_3_–A4, ^13^C_3_–T, D8-17OHP; 0.1 ng) and extracted on a supported liquid extraction SLE400 plate by diluting samples 1:1 (v/v) with 0.1% formic acid in water (v/v) and eluting with 98:2 dichloromethane/isopropanol (v/v; 1.8 mL). Samples were reduced to dryness under a nitrogen gas stream and resuspended in water/methanol (100 μL; 70:30 v/v). Liquid chromatography separation was performed by injecting (20 μL) onto a Kinetex C18 (150 x 2.1 mm; 2.6 µm) column fitted with a KrudKatcher, held at 40^o^C, using a gradient of mobile phase A – water with 50 μmol/L ammonium fluoride and mobile phase B – methanol with 50 μmol/L ammonium fluoride and a flow rate of 0.3 mL/min over 16 mins, starting at 50 % B for 4 mins, rising to 75 % B over 5 mins, rising to 100% B over 1 min, held for 2 mins, then returning to 50 % B over 0.1 mins and equilibrating for 3.9 mins. The flow was diverted to waste from 0–2 mins and 11–16 mins. Separation was followed by ionisation in an IonDrive Turbo V source in electrospray ionisation positive ion mode using multiple reaction monitoring (MRM) on a QTrap 6500+ (Sciex, UK) held at 600^o^C, 5.5 kV with curtain gas at 30, collision gas medium, ion source gas one and two at 40 and 60 arbitrary units respectively and an entrance potential of 10 V.

MRM parameters for each steroid were as follows, quantitative ion followed by qualitative ion, with declustering potential, collision energy and collision exit potential in parenthesis: cortisol *m/z* 363.1 -> 121.2 (66, 31, 12) *m/z* 363.1 -> 91.0 (76, 83, 10); D8-corticosterone *m/z* 355.3 -> 128.1 (37,45,14) *m/z* 355.3 -> 125.0 (29, 56, 14); androstenedione *m/z* 287.1 - > 97.0 (61, 27, 14), *m/z* 287.1 -> 78.9 (61, 67, 10); testosterone *m/z* 289.1 -> 97.0 (101, 29,12), m/z 289.1 -> 109.2 (101, 31,6) 17α-hydroxyprogesterone *m/z* 333.1 -> 109.1 (66,31,12) *m/z* 333.1 -> 96.9 (66, 29, 12). MRM parameters for each internal standard were as follows: D4-cortisol *m/z* 367.2 -> 121.1 (80, 29, 16); ^13^C_3_–A4 *m/z* 290.2 -> 100.1 (31, 27, 12); ^13^C_3_–T *m/z* 292.1 -> 100.0 (96, 29, 12); [^2^H]_8_-17OHP *m/z* 339.2 -> 96.9 *m/z* (66, 29, 12).

Retention times for each analyte were cortisol (3.5 mins), D8-corticosterone (5.2 mins), androstenedione (6.9 mins), testosterone (7.6 mins), 17α-hydroxyprogesterone (8.0 mins); with internal standards D4-cortisol (3.4 mins), ^13^C_3_–A4 (6.8 mins), ^13^C_3_–T (7.6 mins), and D8-17OHP (8.0 mins).

LC-MS/MS data was evaluated using MultiQuant 3.0.3 software (Sciex, Macclesfield, UK), using D4-cortisol as internal standard for both cortisol and D8-corticosterone, ^13^C_3_–A4 for androstenedione, ^13^C_3_–T for testosterone and D8-17OHP for 17α−hydroxyprogesterone. Lines of regression were fitted with a weighting of 1/x or 1/x^2^, with regression coefficients r>0.99.

**Validation Procedure**

Standard curves were prepared (steroid ranges described in Analytical Methodology and indicated in Supplementary Tables 1 and 2) on three separate days. Analytical standards were prepared in water. On each day, DC Mass Spect Gold serum (200 µL) was enriched with steroid analytes (amounts as indicated in Tables S1 and S2) and internal standards and extracted as described above to generate six replicate samples of the value equivalent to each of the lower limit of quantitation and typical low, medium and high values.

**Calibration standard curves**

Lines of regression were fitted with a weighting of 1/x for all compounds, apart from D8-corticosterone which used a weighting of 1/x^2^, yielding regression coefficients within the acceptable range of r>0.99 (Table S1).

**Accuracy and Precision of calibration standards**

The amounts of analytes in samples representing the lower and upper limits of quantitation were back calculated from the associated calibration line, on each of the three occasions. Table S1 shows the mean values of the n=3 analytical runs for precision (expressed as % relative standard deviation) and accuracy (expressed as % nominal value).

**Accuracy and Precision of matrix enriched quality controls**

The amounts of analytes in representative quality control samples calculated from the associated calibration lines, on each of the three occasions. Table S2 shows the mean values for precision (expressed as % relative standard deviation) and accuracy (expressed as % nominal value) for intra-assay assessment (n=6 samples analysed on the same day) and inter-assay assessment (3 replicate batches of 6 samples prepared and analysed on 3 different days). All outcomes were within the desired criteria set by ICH^2^.

**Recovery and Matrix Effects**

Gold serum ((200 µL), n=6) was extracted with addition of certified steroid standards spiked at the following concentrations: cortisol (200 ng/mL), D8-corticosterone (400 ng/mL), testosterone (20 ng/mL), androstenedione (20 ng/mL) and 17α-hydroxyprogesterone (100 ng/mL) before (pre-spike) and after extraction (post-spike). Unextracted standards of the sample amounts were also analysed (n=6). The recovery was assessed by comparing the peak area of analytes on pre vs post spiked samples. The matrix effect was assessed by comparing the peak areas of post-spike samples to those that were unextracted (Table S1). The recovery for all analytes fell within the desired criteria^2^ and the matrix effect was acceptable for all analytes except androstenedione where a great influence was observed. However, this analyte passed all other validation criteria for quantitation, but may be more subject to variability between different cohorts of subjects.

**References**

1. Devine K, Russell CD, Blanco GR, Walker BR, Homer NZM, Denham SG, Simpson JP, Leavy OC, Elneima O, McAuley HJC, Shikotra A, Singapuri A, Sereno M, Saunders RM, Harris VC, Houchen-Wolloff L, Greening NJ, Lone NI, Thorpe M, Greenhalf W, Chalmers JD, Ho LP, Horsley A, Marks M, Raman B, Moore SC, Dunning J, Semple MG, Andrew R, Wain LV, Evans RA, Brightling CE, Kenneth Baillie J, Reynolds RM, Investigators IC & Group P-CSC. Plasma steroid concentrations reflect acute disease severity and normalise during recovery in people hospitalised with COVID-19. *Clin Endocrinol (Oxf)* 2024 **100** 317-327.

2. European Medicine Agency. ICH M10 on bioanalytical method validation - Scientific guideline. EMA/CHMP/ICH/172948/2019. <https://www.ema.europa.eu/en/ich-m10-bioanalytical-method-validation-scientific-guideline>.
