# Supplementary material for: Proof of concept for a superior therapeutic index of corticosterone compared with hydrocortisone in patients with congenital adrenal hyperplasia
Source: Eur J Endocrinol. 2024 Nov 15;191(6):535–44. doi: 10.1093/ejendo/lvae144 (PMC11606648; doi:10.1093/ejendo/lvae144)
Supplement: lvae144_Supplementary_Data [file lvae144_supplementary_data.zip › eje-24-0250-File008.docx]

***Table S1 Inter-assay accuracy and precision of analysis of standards at upper and lower limits of quantitation***

*Recovery (%) was assessed by comparing the peak area of analytes which were spiked pre- or post-extraction using concentrations of 200 ng/mL (cortisol), 400 ng/mL (D8-corticosterone), 20 ng/mL (testosterone and androstenedione) and 100 ng/mL (17α-hydroxyprogesterone). The effect of matrix was assessed by comparing the peak area of post-spike samples to those that were unextracted. LLOQ = Lower limit of Quantitation, ULOQ = Upper Limit of Quantitation. RME = Relative Mean Error, RSD =Relative Standard Deviation.*

| (n=3) | **Cortisol** | **D8-Corticosterone** | **Testosterone** | **Androstenedione** | **17α-Hydroxyprogesterone** |
| --- | --- | --- | --- | --- | --- |
| **LLOQ (ng/mL)** | 1.25 | 2.50 | 0.125 | 0.125 | 5.00 |
| **Inter-assay precision (% RSD)** | 6.1 | 4.0 | 6.5 | 5.2 | 5.0 |
| **Inter-assay accuracy (% RME)** | 110.5 | 96.9 | 112.4 | 112.7 | 101.8 |
| **ULOQ (ng/mL)** | 250.00 | 500.00 | 25.00 | 25.00 | 125.00 |
| **Inter-assay precision (% RSD)** | 3.3 | 3.3 | 2.5 | 2.2 | 8.3 |
| **Inter-assay accuracy (% RME)** | 100.7 | 95.2 | 102.6 | 101.8 | 96.0 |
| **Regression Coefficient (range)** | 0.9986 - 0.9992 | 0.9973 - 0.9981 | 0.9978 - 0.9995 | 0.9984 - 0.9992 | 0.9949 – 0.9995 |
| **Intercept (range)** | 0.0002 - 0.0153 | 0.0030 - 0.0124 | 0.0052 - 0.0066 | 0.0061 - 0.0071 | 0.0300 - 0.3945 |
| Recovery and Matrix effects (n=6) | | | | | |
| **Recovery (%)** | 72 | 73 | 62 | 59 | 85 |
| **Matrix (%)** | 113 | 125 | 120 | 148 | 112 |
