# Supplementary material for: Proof of concept for a superior therapeutic index of corticosterone compared with hydrocortisone in patients with congenital adrenal hyperplasia
Source: Eur J Endocrinol. 2024 Nov 15;191(6):535–44. doi: 10.1093/ejendo/lvae144 (PMC11606648; doi:10.1093/ejendo/lvae144)
Supplement: lvae144_Supplementary_Data [file lvae144_supplementary_data.zip › eje-24-0250-File009.docx]

***Table S2 Intra and Inter-assay accuracy and precision of analysis of quality control serum spiked representing LLOQ, low, medium and high amounts of analytes***

*LLOQ = Lower limit of Quantitation. Accuracy defined by % RME = Relative Mean Error, Precision defined by % RSD =Relative Standard Deviation, QC = Quality Control.*

|  | **LLOQ QC** | | | |  | **Low QC** | | |  | **Mid QC** | | |  | **High QC** | | |
| --- | --- | --- | --- | --- | --- | --- | --- | --- | --- | --- | --- | --- | --- | --- | --- | --- |
| **Analyte** | **Calibration Range (ng/mL)** | **Amount**  **(ng/mL)** | **%RME** | **%RSD** |  | **Amount (ng/mL)** | **%RME** | **%RSD** |  | **Amount**  **(ng/mL)** | **%RME** | **%RSD** |  | **Amount**  **(ng/mL)** | **% RME** | **%RSD** |
|  | **Intra-assay comprising n=6 replicate samples analysed on the same day** | | | | | | | | | | | | | | | |
| **Cortisol** | 1.25 - 250 | 1.25 | 94.8 | 9.8 |  | 3.75 | 99.8 | 2.4 |  | 100 | 103.5 | 7.2 |  | 200 | 104.3 | 4.1 |
| **D8-Corticosterone** | 2.5 - 500 | 2.5 | 87.3 | 10.2 |  | 7.5 | 89.4 | 1.6 |  | 200 | 88.2 | 8.3 |  | 400 | 89.3 | 4.9 |
| **Testosterone** | 0.125 - 25 | 0.125 | 108.7 | 6.8 |  | 0.375 | 100.4 | 3.0 |  | 10 | 98.2 | 7.8 |  | 20 | 105.0 | 5.0 |
| **Androstenedione** | 0.125 - 25 | 0.125 | 111.3 | 6.6 |  | 0.375 | 101.3 | 2.0 |  | 10 | 98.9 | 7.5 |  | 20 | 105.6 | 4.7 |
| **17α-Hydroxyprogesterone** | 5 - 125 | 5.0 | 95.7 | 8.7 |  | 15.0 | 102.6 | 7.7 |  | 50 | 111.2 | 2.3 |  | 100 | 96.3 | 7.4 |
|  | **Inter-assay comprising n=6 replicate samples analysed on each of 3 days** | | | | | | | | | | | | | | | |
| **Cortisol** | 1.25 - 250 | 1.25 | 94.5 | 10.9 |  | 3.75 | 95.7 | 5.5 |  | 100 | 98.8 | 8.7 |  | 200 | 101.7 | 5.3 |
| **D8-Corticosterone** | 2.5 - 500 | 2.5 | 92.2 | 10.5 |  | 7.5 | 98.8 | 8.2 |  | 200 | 95.9 | 10.5 |  | 400 | 96.7 | 8.3 |
| **Testosterone** | 0.125 - 25 | 0.125 | 106.3 | 7.1 |  | 0.375 | 98.9 | 2.7 |  | 10 | 96.0 | 7.4 |  | 20 | 102.9 | 4.7 |
| **Androstenedione** | 0.125 - 25 | 0.125 | 107.0 | 7.0 |  | 0.375 | 99.3 | 3.1 |  | 10 | 96.2 | 7.5 |  | 20 | 103.0 | 5.0 |
| **17α-Hydroxyprogesterone** | 5 - 125 | 5.0 | 99.3 | 7.5 |  | 15.0 | 98.7 | 6.2 |  | 50 | 102.8 | 11.0 |  | 100 | 92.5 | 7.3 |
